# Supplementary material for: Premature translation of the Drosophila zygotic genome activator Zelda is not sufficient to precociously activate gene expression
Source: G3 (Bethesda). 2022 Jul 25;12(9):jkac159. doi: 10.1093/g3journal/jkac159 (PMC9434156; doi:10.1093/g3journal/jkac159)
Supplement: jkac159_Supplemental_Figures [file jkac159_supplemental_figures.pdf]

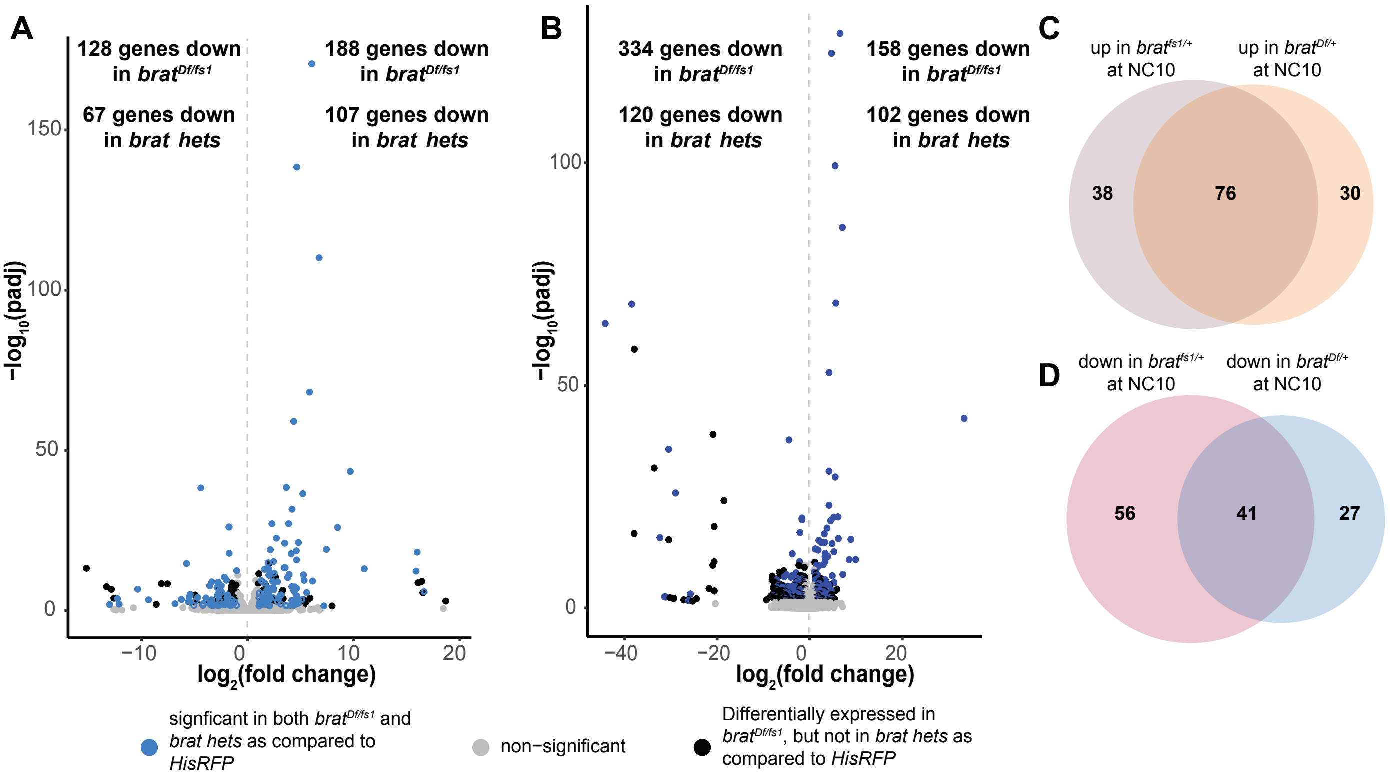


**Figure S1: Embryos heterozygous for *brat* mut alleles display differential gene expression.** A. Volcano plot of differentially expressed genes (L2FC >1, padj <0.05) in NC10 embryos laid by *brat*$^{Df/fs1}$ mothers as compared *HisRFP* NC10 embryos. Genes that are also misexpressed in NC10 embryos laid by mothers heterozygous for a *brat* mutant allele (*brat het*) as compared to *HisRFP* cotrols are in blue. All other significantly differentially expressed genes are in black and non-significant genes are in gray. B. Volcano plot of differentially expressed genes (L2FC >1, padj <0.05) in *brat*$^{Df/fs1}$ oocytes compared *HisRFP* oocytes. Genes that are also misexpressed in *brat het* oocytes compared to *HisRFP* cotrols are in blue. All other significantly differentially expressed genes are in black and non-significant genes are in gray. C. Overlap of individual genes that show increased expression in NC10 embryos laid from either *brat*$^{fs1/+}$ or *brat*$^{Df/+}$ heterozygous mothers. D. Overlap of individual genes that show decreased expression in NC10 embryos laid from either *brat*$^{fs1/+}$ or *brat*$^{Df/+}$ heterozygous mothers.

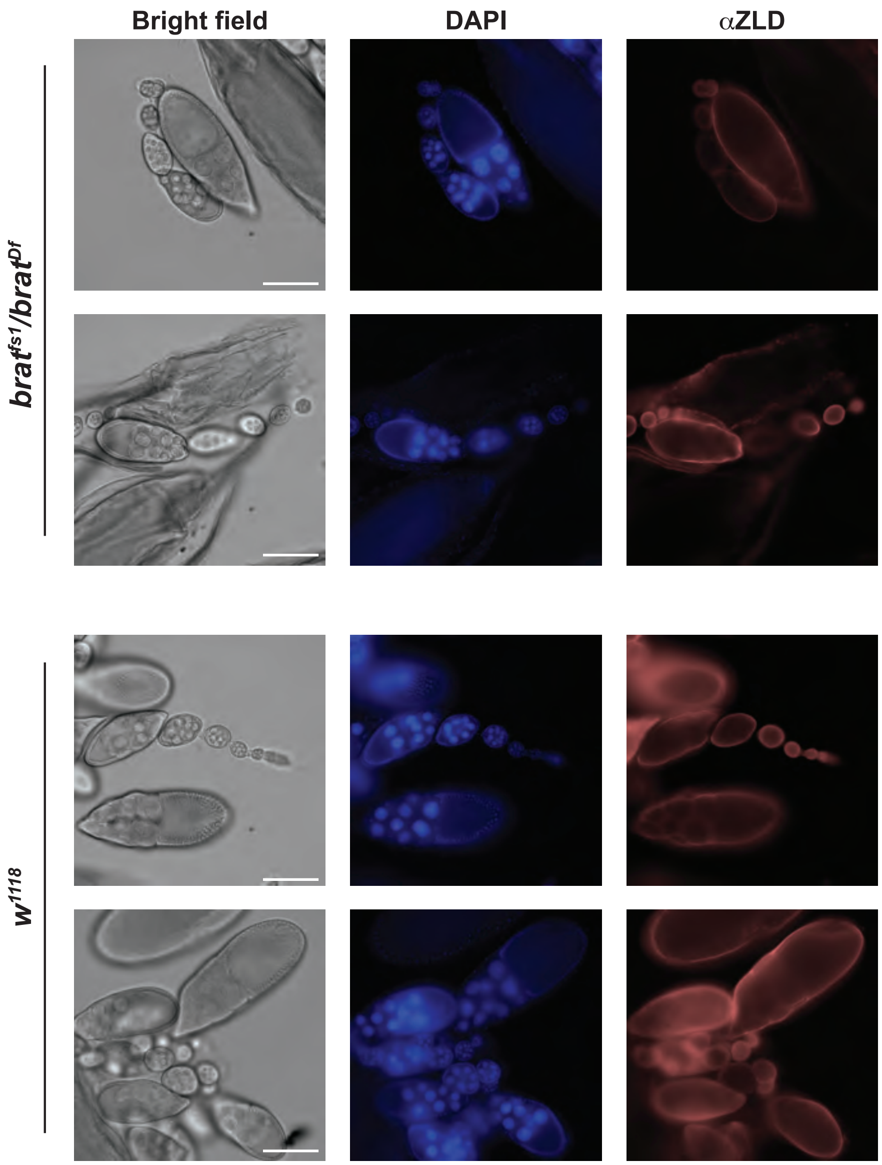


**Figure S2: ZLD protein is not evident in *brat*$^{fs1}$/*brat*$^{Df}$ or wild-type ovaries.** Immunostaining with anti-ZLD antibody failed to detect any ZLD protein in the nurse cells or developing oocytes in control or *brat*-mutant ovaries. Scale bar 100 μm. All images are at the same magnification.

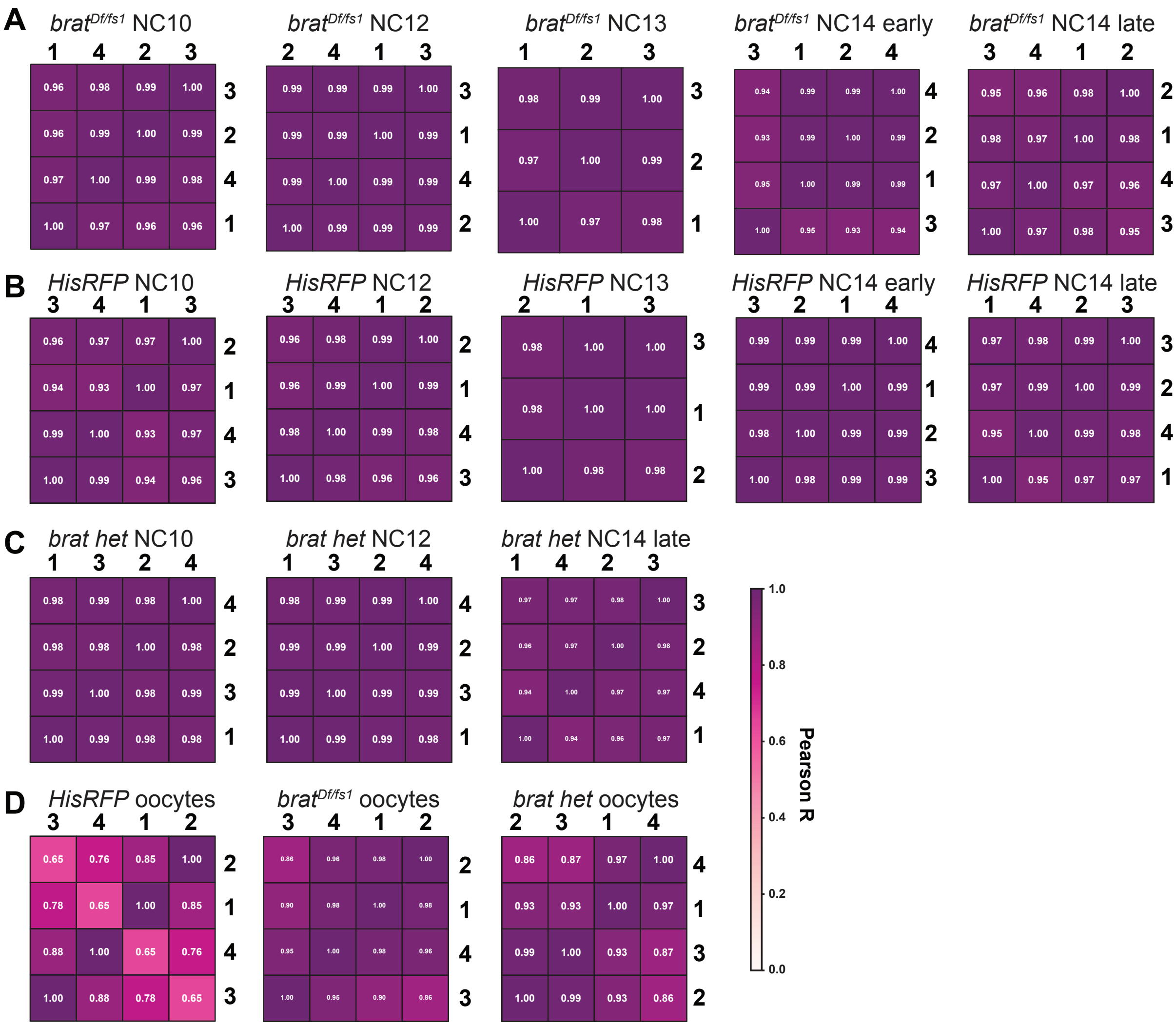


**Figure S3: Single-embryo and single-oocyte replicates are highly correlated.** A-D. Correlation plots of replicates from RNA-seq experiments in *brat*$^{Df/fs1}$ NC10-NC14 embryos (A), *HisRFP* NC10-NC14 embryos (B), *brat het* NC10-NC14 embryos (C) and oocytes (D). The color of each square represents the Pearson R coefficient. Replicates numbers are listed.
