# Supplementary material for: Premature translation of the Drosophila zygotic genome activator Zelda is not sufficient to precociously activate gene expression
Source: G3 (Bethesda). 2022 Jul 25;12(9):jkac159. doi: 10.1093/g3journal/jkac159 (PMC9434156; doi:10.1093/g3journal/jkac159)
Supplement: jkac159_Supplemental_Table_S1 [file jkac159_supplemental_table_s1.zip › jkac159_Supplemental_Table_S1_legend.pdf]

Table S1: Lists of differentially expressed genes. Lists of differentially expressed genes at each time point collected (late stage oocyte, NC10, NC12, NC13, early NC14 and later NC14) between the *brat* mutant and *HisRFP* embryos including the $\log_2$(foldchange,) p value, adjusted p value, MZT class, whether or not the gene is bound by ZLD, whether or not the gene is bound by BRAT, whether or not the gene was upregulated in Laver *et al.* 2015, the zygotic class, whether or not the gene was considered zygotically expressed by Strong *et al.* 2020 and whether or not the gene determined to be differentially expressed using the criteria $\log_2$(fold change) >1, padj <0.05. Comparisons between the *brat* hets and *HisRFP*, and clusters from Figure 5A and classes from Figure 5C are also included.
